# The Orphan Working School

**Published:** 1918-01-19

**Authors:** F. J. Frankau

**Affiliations:** 9 Lancaster Road, Belsize Park, London, N.W.


### THE EDITOR'S LETTER-BOX.

### The Orphan Working School.

*To the Editor of* The Hospital.

Sir,—Under the heading "Hospital and Institutional Results" in your to-day's issue you criticise the accounts of the Orphan Working School. Apparently you have confined your attention to the "Income and Expenditure Account," on pp. 14, 15. If you will turn to pp. 18, 19, you will find a full analysis. The items for what may be called provisions and soap, on the lower part of p. 18, with the column "Groceries," on p. 19, come to £4,233 odd—the total of "Housekeeping" in the Income and Expenditure Account in the column of it headed "Maitland Park" and "Margate." Possibly an academical improvement might be effected by adding the words "Heating and Lighting" to the description of the analysis; but the columns showing cost of gas and electricity and of coal and coke obviously are details of the "Heating and Lighting" item in the Income and Expenditure Account.

It is quite easy to compare our expenditure on any item with that of other like institutions. I only have the London Orphan School, Watford, accounts available at the time of writing; but theirs, like those of most similar charities, are practically "uniform" with ours, if you will only look at our analysis.

Without making invidious distinctions, I venture, as chairman for many years of our house committee, to say that we are as economically managed as any similar institution which treats its children as well as we do, and as can be readily seen even from the accounts you criticise.—I am, yours obediently, F. J. Frankau.

9 Lancaster Road, Belsize Park, London, N.W.,
January 12, 1918.

[We thank Mr. Frankau for drawing our attention to the analysis of the figure stated in the Income and Expenditure Account in respect of "Housekeeping"; we should have found these details sooner if there had been a direct reference. There is nothing inherent to the conditions of an orphanage which forbids the use of the Uniform System, and the accounts in question would be much clearer were such a system to be adopted. Except in respect of the educational side the income and expenditure will naturally fall under the headings chiefly used. We have no reason to think that the Orphan Working School, which is an excellent institution, is not run on economical lines, but we would be better able to judge if our suggestions were carried out.—Ed. The Hospital.]

